# A case report and literature review on a rare subtype of triple-negative breast cancer in children

**DOI:** 10.1186/s12887-023-04286-6

**Published:** 2023-09-29

**Authors:** Lexiang Shi, Yinan Zhang, Jingcheng Wu, Jinping Li, Junzhao Zhu, Youbo Xu, Nie Li, Qin Li, Wanli Zhang

**Affiliations:** 1 Xi’an International Medical Center Hospital, Xi’an, China; 2 Department of Pediatric Surgery, Huzhou Maternity & Child Health Care Hospital, No. 2 East Street, Wuxing DistrictZhejiang Province, Huzhou City, China; 3 Mudanjiang Medical University, Mudanjiang, China

**Keywords:** Triple-negative breast cancer, Secretory breast cancer, Children

## Abstract

**Background:**

Triple-negative breast cancer (TNBC) is a type of breast tumor with a poor prognosis because it lacks or expresses low levels of estrogen receptor (ER), progesterone receptor (PR), and human epidermal growth factor receptor 2 (HER-2). TNBC is more common in middle-aged and older women, and cases of TNBC in children are rarely reported. This is the only case of childhood SBC in our hospital in more than 70 years, and the disease is extremely rare internationally. We analyzed and studied the disease and TNBC from both clinical and pathological aspects and found that SBC is very different from TNBC.

**Case presentation:**

We report a case of secretory breast cancer (SBC), a subtype of TNBC, in an 8-year-old girl from our institution. The child presented with a single mass in the left breast only, with no skin rupture and no enlargement of the surrounding lymph nodes. The child underwent two surgeries and was followed up for one year with a good prognosis.

**Conclusions:**

SBC is highly prevalent among the multiple pathological types of pediatric breast cancer. Almost all pediatric SBC patients are characterized by the ETV6-NTRK3 fusion gene, which has a good prognosis and a 10-year survival rate of more than 90% when compared with other TNBC subtypes. According to the patient, we performed local mass resection and a postoperative pathological diagnosis of SBC (a subtype of BL-TNBC). The TNBC case had a good prognosis and differed from basal TNBC in several aspects, including clinical presentation, treatment, and prognosis. It is necessary to exclude SBC from BL-type TNBC, enhance understanding of the disease, and individualize the treatment plan, so as to avoid medical errors.

## Background

As a common malignant tumor in humans, breast cancer has jumped to the top of malignant tumors in terms of prevalence, and it is the leading cause of female malignant tumors [1]. Triple-negative breast cancer(TNBC) is a subtype of breast cancer, which accounts for approximately 15–25% of breast cancer cases and is characterized by the absence or reduction of estrogen receptor (ER), progesterone receptor (PR), and human epidermal growth factor receptor 2 (HER-2) [2]. The criteria for ER/PR-negative are met if there is less than 1% expression of those receptors within the tumor. A tumor is considered HER2-negative if the immunohistochemistry result is 0, 1 + or 2 + , and FISH negative. TNBC has a high recurrence rate, a high metastatic potential, and a short overall survival, with some reports indicating that patients with recurrent metastatic TNBC have an overall survival of only 13–18 months [3]. secretory breast cancer(SBC) is more common in middle-aged women [4, 5]. SBC accounts for less than 0.15% of breast cancers, with a higher incidence in adults than in children and that in the female than in the male, with previous literature showing a ratio of 6:1 and a recent National Cancer Data Base(NCDB) review demonstrating a ratio of 31:1 [6]. The pathogenesis is closely related to genomic instability and mutations [7]. A study by Min Ji Song [8] analyzed the exome sequencing data of three groups of SBCs, totaling 1105 somatic cells, and identified 1,046 somatic mutations. Among the 44 genes detected, 11 mutated genes of SBC (KIAA2012, SUCLG2, SEMA3G, KLHL18, FOXB2, KMT2D, DHX38, CERS4, CD209, AP3D1, HELZ2) differed from those of TNBC. The prognosis for children with SBC is good. There is no consensus on the optimal treatment strategy for SBC. Therefore, it makes sense to report each case to provide treatment experience.

## Case presentation

**Fig. 1:**
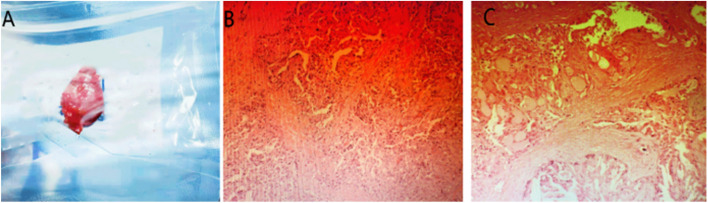
**A**: Tumour. **B** and **C**: Postoperative pathological results of the tumour under the microscope(secretory vacuoles can be seen)

An 8-year-old girl was presented to Red Flag hospital with a progressive enlargement of her right breast mass for 4 months. She had undeveloped breasts, pubic/axillary hair development at Tanner 1 stage, no menstrual flow, no birthmarks of any kind, no areas of mucosal pigmentation; and no findings suggestive of cancer susceptibility syndrome. There was a hard mass about 1.5 cm in diameter under the right nipple, with clear borders and good mobility. Preoperative ultrasonography showed a hypoechoic lesion near the right nipple, measuring about 1.4 × 0.8 cm, with clear borders, regular morphology, and obviously blood flow signal. The patient has no family history of breast cancer or other types of cancers (ovarian cancer, colon cancer, etc.). The presence of gastrointestinal polyps could not be determined because no relevant tests were performed. The patient underwent local excision of the mass. The tumor was white to gray-red tissue measuring 1.6 × 1.4 × 0.7 cm, with a solid gray-white cut surface. Histological findings showed a dominant papillary structure, as well as tubular pink or pale pink cells with secretory vesicles and interstitial powdery secretions. Immunohistochemical results showed ER few cells ( +), PR) (-), HER-2 (-), and Ki-67 less than 10% ( +). A diagnosis of SBC was made. The child underwent a second extended excisional scope surgery with an anterior lymph node biopsy. No tumor cells were found in the residual tumor cavity around the incision margin or in the anterior lymph node biopsy. The child did not undergo next-generation breast cancer multigenomic testing but fluorescence in situ hybridization (FISH) for the ETV6 gene, which revealed the presence of the ETV6-NTRK3 fusion gene. The patient presented with features of a benign breast tumor. The tumor was small in size (less than 2.0 cm), with clear borders, no adhesion to surrounding tissues, no nipple discharge, and no axillary lymph node metastasis. There was no obvious adhesion or tumor malignant tendency during the operation. Based on the therapeutic precedents documented in pertinent scholarly literature, it is not necessary for the patient to undergo radiotherapy, chemotherapy, immunotherapy, or targeted therapy subsequent to the surgical procedure. (The tumor and postoperative pathological images were shown in Fig. 1).

## Discussion

In recent years, TNBC has become a hot spot for clinical research in recent years due to an increase in incidence and a younger age group [9]. Studies have shown that deletion or mutation of breast cancer1/2(BRCA1/2) is closely associated with the development of TNBC. The term "homologous recombination defect" (HRD) refers to a situation in which the homologous recombination (HR) process is unavailable or impaired [10, 11]. In this case, DNA repair is more prone to errors, leading to genomic instability. Breast cancer susceptibility gene 1/2 (BRCA1, 2) is an oncogene that maintains genomic stability by playing a key role in DNA repair, cell cycle arrest, and transcriptional control. BRCA1 and BRCA2 play a role in DNA double-strand break (DSB) repair through the HR process, with BRCA1 guiding repair toward error-free HR [12]. BRCA1/ 2 losses of function lead to HRD [13]. Ki67 is significantly associated with the proliferation level of tumor cells and can predict the prognosis of TNBC [14]. Recent studies have shown that programmed death-ligand 1 (PD-L1) and purine/pyrimidine endonuclease 1 (APE1) play a role in the development of TNBC. It is hypothesized that APE regulates PD-L1 expression, promotes greater metastatic and invasive capacity of tumor cells and further participates in tumor immune escape [15]. Therefore, the emergence of immune checkpoint therapies may be a future research direction. SBC has a characteristic t (12:15) homozygous transition that produces an ETV6-NTRK3 gene fusion. The resulting chimeric tyrosine kinase produced activates the Ras-Mek1 and PI3K-Akt pathways, which are essential for the growth and survival of mammary cells, and have potent transforming activity on fibroblasts and mammary duct epithelial cells. This ultimately leads to the emergence of SBC [16]. The current general consensus for SBC is to perform FISH fusion gene testing, so BRCA, PDL-1, and APE1 testing are not performed in patients. TNBC is widely thought to be highly malignant and has a poor prognosis, but a large body of literature shows that TNBC is heterogeneous and varies greatly in terms of pathological features, biological behavior and gene expression profiles [2, 17–19]. Therefore, it is important to understand the classification of TNBC in clinical practice. TNBC can be classified into several subtypes based on their biological characteristics. Chen Lin [20] found that the classification of luminal androgen receptor (LAR), basal-like (BL), mesenchymal (MES), and immunomodulatory/basal-like immune activation (IM/BLIA) was repeatedly mentioned in several TNBC classifications and is a relatively recognized one. JoensuuH [21] classified TNBC into BL and non-BL types, with BL type accounting for 80%, having a high degree of malignancy and a poor prognosis compared to the non-BL type. BL-TNBC is further divided into BL1 and BL2 types, which are characterized by overexpression of cell cycle-related genes and DNA damage-response genes [22] and exhibit a strong proliferative capacity through these features. BL-1 has a higher value-added rate than other subtypes of TNBC [23], indicating active tumor cell proliferation and poor prognosis. Breast cancer in children [24] can be diagnosed by clinical examination, radiological/imaging examinations (including mammography, magnetic resonance imaging (MRI), ultrasonography, etc.), and immune histopathological examination. TNBC is diagnosed by using intraoperative rapid frozen section pathology and postoperative pathological findings. The primary treatment for TNBC is surgery combined with neoadjuvant chemotherapy [25]. As different histological subtypes of TNBC vary greatly in clinical presentation, treatment, and prognosis; histological classification can help scientists to develop the best-individualized treatment approach. Platinum-based drugs and PARP inhibitors [16] are effective in the treatment of TNBC of BL-1 type.

According to the worldwide classification of intrinsic subtypes, SBC is classified as basal-like TNBC [26]. However, the profile of SBC is very different from that of TNBC. SBC accounts [27] for less than 0.15% of breast cancers but is more prevalent in children with a family history of cancer. In children, SBC is usually solitary, firm, well-defined, less than 2.0 cm in diameter, mobile, slow-growing, and mostly with no lymph node metastasis. When SBC is combined with other breast diseases (intraductal papillary carcinoma or fibroadenoma), the mass typically shows signs of malignant breast cancer, such as spillage, poorly defined borders, poor mobility, [28] and rapid mass growth. On ultrasonography, they are mostly well-defined cystic masses or solid nodules with isoechoic or hypoechoic. The microscopic histologic pattern of SBC contains four main morphologies: microcystic, solid lamellar, tubular and papillary structures, with most cases containing all four morphologies in varying proportions, while a few cases are completely dominated by a single histologic pattern. Early diagnosis of SBC in children is difficult. Conventional imaging is shy of diagnosis. However, recent studies suggest that elastography scoring examinations [29] may offer a new method for the early diagnosis of SBC in children. Elastography is an advanced modality of ultrasound imaging that leverages variations in tissue stiffness for diagnostic purposes, effectively differentiating between soft and hard tissues. This technique is particularly instrumental in the detection of malignant lesions in organs such as the thyroid and breast. This technique is particularly instrumental in the detection of malignant lesions in organs such as the thyroid and breast. The elastography scoring standard includes a total of five points: the lesion is combined or not with green, the lesion and its surroundings are blue, 5 points, a little green in the lesion or blue overall, 4 points, blue and green in the lesion. The scale is equal to three points, with green around the lesion and blue inside, as two points, and the lesion is basically or completely green as one point. Score 4 or higher indicates malignancy, and Score 3 indicates benign. The surgical approach to SBC in children is under discussion. For SBC children with tumors less than 2.0 cm and good clinical performance, local mass excision and breast-conserving surgery can be performed, followed by sentinel lymph node biopsy [30]. SBC has good prognosis, with a 10-year survival rate higher than 90% [31]. For children with tumor diameter greater than 2.0 cm, rupture, nipple discharge and lymph node metastasis, modified radical mastectomy or radical mastectomy for breast cancer should be performed. Tyrosinase and RAS inhibitors [32] can be used as therapeutic agents in children who have a significant malignant tendency or even metastasis. This is the first case of SBC in a child in our hospital. The attending surgeon performed a local tumor resection by making a minimally invasive incision from the outside of the breast. During the operation, the tumor was found to adhere slightly to the breast but did not infiltrate the surrounding tissue. Unfortunately, no interpretative rapid pathological examination was performed. The lesson for us is that doctors must always be serious and responsible. Before starting the operation, all the conditions that may occur during the operation should be evaluated and appropriate treatment solutions should be provided for patients. Children with tumors at special sites should be treated cautiously and the possibility of malignant results should be considered during the operation. A quick pathological examination is required during the operation. If the results suggest that it is a malignant tumor, the scope of resection and lymph node dissection should be expanded to ensure the child’s safety to avoid physical, psychological, and financial harm from a second operation.

## Conclusions

Although SBC is classified as a BL-1 type of TNBC, it differs significantly from BL-TNBC in terms of pathogenesis, clinical presentation, treatment, and prognosis. Therefore, it is extremely important to exclude SBC from BL-TNBC and to select the optimal individualized treatment plan. Some scholars [16, 33] believe that distinguishing SBC from BL-TNBC was necessary to avoid misdiagnosis and mistreatment. Childhood breast cancer is mainly associated with genetic mutations. Among the numerous subtypes of breast cancer, Secretory Breast Carcinoma (SBC) is the most prevalent in children, despite its relative rarity. Notably, SBC typically presents an excellent prognosis [34]. The case of our current patient aligns with these findings reported in the literature. We concur with these observations. The purpose of this study is to supplement the SBC sample database to inform epidemiological studies of the disease and to improve pediatric surgeons' management of pediatric breast tumor disease. This is the first report to question the classification of TNBC from a clinical and pathological perspective. Despite these differences, SBC has so far been classified as a BL-1 subtype of TNBC; whether this is due to TNBC heterogeneity or other factors remains to be further studied and confirmed.

## Data Availability

All data generated or analyzed during this study are included in this article and its supplementary information files.

## Abbreviations

TNBC: Triple-negative breast cancer
ER: Estrogen receptor
HER-2: Human epidermal growth factor receptor 2
BL: Basal-like
PR: Progesterone receptor
PD-L1: Programmed death ligand 1
CEA: Carcinoembryonic antigen
APE: Apurine/pyrimidine endonuclease 1
DSBs: DNA double-strand breaks
SSB: Single-Strand Break
